# Annual acknowledgement of manuscript reviewers

**DOI:** 10.1186/1472-6785-14-2

**Published:** 2014-02-27

**Authors:** Simon Harold

**Affiliations:** 1 BioMed Central, Floor 6, 236 Gray's Inn Road, London WC1X 8HB, UK

## Abstract

**Contributing reviewers:**

The editors of BMC Ecology would like to thank all of our reviewers who have contributed to the journal in volume 13 (2013).

## 

Peter Adamik

Czech Republic

Walter Adey

USA

Deepa Agashe

India

Harry P Andreassen

Norway

A. Marcia Barbosa

Portugal

Matthew Bracken

USA

Christian Braendle

France

Francesca Cagnacci

Italy

Richard Caners

Canada

Amélie Cantarel

France

Samuel Caro

Belgium

Lauren Carrington

Viet Nam

Pilar Castro-Diez

Spain

Dominic Chaloner

USA

Melody Susan Clark

UK

Olivier Clerck

Belgium

Aurélie Coulon

France

Julien Cucherousset

France

Catherine Cullingham

Canada

Vivian Cumbo

Australia

Ellen Decaestecker

Belgium

Ed Dewalt

USA

Weixin Ding

China

Carsten Dormann

Germany

Gilberto Dos-Santos

USA

Jeremie Fant

USA

James Farlow

USA

Richard Fautley

UK

Hélène Fréville

France

John Fryxell

Canada

Jean-François Le Galliard

France

Jessica Garb

USA

Julien Gasparini

France

Christy Geraci

Iraq

Cara Gibson

USA

Matthew Gilg

USA

Osnat Gillor

Israel

Jeff Graves

UK

Daniel Gruner

USA

Alessia Guggisberg

Switzerland

Anton Güntsch

Germany

Rob Guralnick

USA

Lumir Gvozdik

Czech Republic

Thierry Hance

Belgium

Mark Hewison

France

Thomas Hladish

USA

Chuan-Kai Ho

Taiwan

M Hodson

UK

Danny Hooftman

UK

Katrine Hoset

Finland

Hans Jacquemyn

Belgium

Norbert Juergens

Germany

Brent Kaiser

Australia

Rebecca Kao

USA

Marco Katzenberger

Portugal

Shamus Keeler

USA

Gabrielle Kelly

Ireland

Katy Klymus

USA

Kevin Kohl

USA

Takashi Kohyama

Japan

Christoph Konrad

Germany

Paul Krushelnycky

USA

Susanne Lachmuth

Germany

Todd Lajeunesse

USA

Martin Lange

Germany

Leigh Latta

USA

Charlotte Lee

USA

Delphine Legrand

France

Tom Little

UK

Nikhil Lobo

Canada

Barbara Mable

UK

Carlos Martín

Spain

Erik Matthysen

Belgium

Stuart Mcdaniel

USA

Raul Medina

USA

James Millington

UK

William Mitchell

USA

Ed Mondor

USA

Stephen Munga

Kenya

Zuzana Münzbergová

Czech Republic

Ephantus J Muturi

USA

Atle Mysterud

Norway

Wolfgang Nentwig

Switzerland

Paul Newton

New Zealand

Martyn Obbard

Canada

Mark O'Donoghue

Canada

Anthony O'Grady

Australia

Arpat Ozgul

Switzerland

Charles Parker

USA

Vincenzo Penteriani

Spain

Silvia Perez-Espona

UK

Ronald Pierik

Netherlands

Lori Qakenbush

USA

Michael Raupach

Germany

Eduardo Rebollar-Tellez

Mexico

Jennifer Rehage

USA

Brys Rein

Belgium

Maxime Réjou-Méchain

France

Luke Rendell

UK

Jonathan Richir

Belgium

Katharina Riebel

Netherlands

Alexander Riedel

Germany

Charles Robbins

USA

Benjamin Roche

France

Hannah Rowland

UK

Jörgen Rudolphi

Sweden

Jacob Russell

USA

Kurt Samways

Canada

Carlos Sanz-Lázaro

Spain

Stéphane Sartoretto

France

Heiko Schmaljohann

Germany

Derek Sikes

USA

Graham Smith

UK

Daniel Sol

Spain

Jay Stachowicz

USA

David Stanley

USA

Kerri Steenwerth

USA

Eleanore Sternberg

USA

Virginie M Stevens

France

Maria Strack

Canada

Jukka Suhonen

Finland

Katalin Szlavecz

USA

Daniel Thornhill

USA

Hans-Hermann Thulke

Germany

Fritz Trillmich

Germany

Katrien Vandepitte

Belgium

Matthew Venesky

USA

Mark Viney

UK

Rita Volkers

Netherlands

Darren Ward

New Zealand

Robert Ward

Australia

John Whitlock

Canada

Craig Williams

Australia

Ed Witkowski

South Africa

Sabine Wollrab

Germany

Yoram Yom-Tov

Israel

Johann Zaller

Austria

Lindsay Zanno

USA

Elena Zvereva

Finland

